# Neurocan expression associates with better survival and viral positivity in Merkel cell carcinoma

**DOI:** 10.1371/journal.pone.0285524

**Published:** 2023-05-05

**Authors:** Marko Salmikangas, Maria Laaksonen, Henrik Edgren, Marco Salgado, Anu Suoranta, Pirkko Mattila, Virve Koljonen, Tom Böhling, Harri Sihto

**Affiliations:** 1 Department of Pathology, Medicum, University of Helsinki and Helsinki University Hospital, Helsinki, Finland; 2 MediSapiens Ltd., Helsinki, Finland; 3 Department of Plastic Surgery, University of Helsinki and Helsinki University Hospital, Helsinki, Finland; 4 Institute for Molecular Medicine Finland, Helsinki, Finland; Kimura Hospital, JAPAN

## Abstract

Merkel cell carcinoma (MCC) is a rare cutaneous neuroendocrine carcinoma that is frequently divided into Merkel cell polyomavirus negative and positive tumors due their distinct genomic and transcriptomic profiles, and disease outcomes. Although some prognostic factors in MCC are known, tumorigenic pathways, which that explain outcome differences in MCC are not fully understood. We investigated transcriptomes of 110 tissue samples of a formalin-fixed, paraffin-embedded MCC series by RNA sequencing to identify genes showing a bimodal expression pattern and predicting outcome in cancer and that potentially could play a role in tumorigenesis. We discovered 19 genes among which *IGHM*, *IGKC*, *NCAN*, *OTOF*, *and USH2A* were associated also with overall survival (all p-values < 0.05). From these genes, NCAN (neurocan) expression was detected in all 144 MCC samples by immunohistochemistry. Increased NCAN expression was associated with presence of Merkel cell polyomavirus DNA (p = 0.001) and viral large T antigen expression in tumor tissue (p = 0.004) and with improved MCC-specific survival (p = 0.027) and overall survival (p = 0.034). We conclude that NCAN expression is common in MCC, and further studies are warranted to investigate its role in MCC tumorigenesis.

## Introduction

Merkel cell carcinoma (MCC) is a rare neuroendocrine carcinoma of the skin, most commonly discovered on sun-exposed skin areas of elderly Caucasians [1]. In the majority of MCCs, integration of the Merkel cell polyomavirus (MCPyV) genome into the host cell genome leads to tumorigenesis. MCC can be divided into two distinct subgroups, virus-negative (VN-MCC) and virus-positive MCCs (VP-MCC), which have distinct genotypes, with prognosis being worse in VN-MCC [2–5].

Genomic profiling studies show that VN-MCCs exhibit frequent *MYC-L* amplifications, *RB1*, *TP53*, *NOTCH1*, and *PIK3CA* gene mutations, high mutation burden, and ultraviolet mutation signature [4,6–8]. However, a high number of tumor-infiltrating lymphocytes and upregulation of antigen presentation and immune response genes are associated with a better survival in MCC and VP-MCC [4,9–13]. Interestingly, viral T antigens of MCPyV target the same genes that are mutated in VN-MCC. A viral sT antigen binds MYCL protein, contributing to cellular transformation and reduction in HLA-I antigen presentation and p53 tumor suppressor activity [14–16]. LT antigen, on the other hand, binds pRb and promotes tumor growth [17,18].

Despite of the shared cancer driver genes, VN-MCC and VP-MCC show a bimodal distribution of tumor mutation burden, mutation signatures, DNA methylation pattern and transcriptome profiles in genomic and transcriptomic studies [4,6,7,19]. We have also reported earlier that tumors from the patients who died for MCC have a distinct gene expression profile when compared with survivors [11]. In addition, MCC forms three molecular clusters based on transcriptome profiling, and on the highest hierarchical level VN-MCCs and VP-MCCs are divided into the two main subtypes [20]. Therefore, in this study, we aimed to identify new bimodally expressed genes that may drive MCPyV-status associated tumorigenesis, explain survival differences, or potentially be utilized as therapy targets in MCC. To this end, we investigated bimodally expressed genes in 110 MCC tissue samples by using SIBER analysis (systematic identification of bimodally expressed genes using RNAseq data) and RNA sequence data that was generated in the previous study [11]. The analysis yielded 19 such genes, including *neurocan* (*NCAN*). NCAN expression was evaluated further in MCC tissue microarray series by immunohistochemistry and its association with clinicopathological factors were investigated.

## Materials and methods

### Sample series

Data on patients diagnosed between 1 January 1979 and 31 December 2013 with MCC in Finland were obtained from the Finnish Cancer Registry, the Helsinki University Hospital files, and the Helsinki Biobank [2,11,21]. Clinical data were extracted from records of hospitals and primary care centers. Data on cause of death and survival were obtained from the Finnish Cancer Registry and the Register Office of Helsinki. Tumor tissue was available in 142 samples to conduct RNA sequencing.

Formalin-fixed, paraffin-embedded (FFPE) tissue blocks were retrieved from pathology archives and the Helsinki Biobank. Tissue microarray (TMA) blocks were constructed from the tissue material in collaboration with the Helsinki Biobank using 1 mm punches with TMA Grand Master (3DHISTECH, Budapest, Hungary). MCPyV status of the primary tumors was assessed with immunohistochemistry (CM2B4 staining for MCPyV LT antigen) and qPCR (MCPyV Large T-antigen primers), as described in detail elsewhere [21].

The study protocol was approved by the Ethics Committee of Helsinki University Hospital and the local review board. The need for informed consent was waived by the Ministry of Health and Social Affairs, which granted permission to collect patient data, and the National Authority for Medicolegal Affairs, which granted permission to collect and analyze tissue samples.

### RNA extraction and RNA sequencing

Two 10-μM sections from FFPE MCC primary tumor samples of a cancer-representative area were used as starting material for the RNA extractions. RNA extraction was performed with a QIAsymphony SP instrument (QIAGEN GmbH, Hilden, Germany) and QIAsymphony RNA extraction kit (cat. no. 931636, QIAGEN GmbH) following the manufacturer’s protocol. If a percentage of tumor cells was less than 50% in the whole tissue section, a cancer-presentative area was scraped from a microscope slide with a scalpel to RNA extraction. The quality and quantity of the extracted RNA were measured using a 2100 Bioanalyzer (Agilent Technologies, CA, USA). The average RNA integrity number (RIN) was 2.0 (range 1.0 to 6.2).

RNA sequencing was conducted at the sequencing unit of the Institute of Molecular Medicine Finland, Technology Centre, as described earlier [11]. Briefly, up to 75.6 ng of RNA was used as a starting material for library preparation, and 1 μl of 1:1000 ERCC RNA spike-in control was added to each sample. RNA sequencing (RNA-seq) library was prepared in 48 sample batches by using QuantSeq 3’ mRNA-seq Library kit (Lexogen, Vienna, Austria, version 015UG009V0221) according to the manufacturer’s instructions. The sequencing was carried out with a single end run. Sample libraries homogeneous in average size and concentration were pooled together with equal molarity and sequenced with 39 samples per lane with high-output mode and v4 chemistry by using a HiSeq 2500 instrument (Illumina, Inc., San Diego, CA, USA), with sequencing read length of 101 bp and allowing one mismatch during demultiplexing. The RNA sequence data were submitted to the SRA database and can be accessed under BioProject PRJNA775071.

### Bioinformatics

The Lexogen QuantSeq 3’ mRNA-seq kit has an integrated data analysis pipeline on the BlueBee Genomics platform (BlueBee Holding BV, Rijswijk, the Netherlands). The BlueBee genomics platform was used to acquire read counts according to the manufacturer’s instructions. The average read count per sample was 6.3 million, with an alignment rate of 57%. After quality control, 111 of the 142 sequenced samples (78%) had an acceptable read count and quality for further processing. One sample was sequenced twice, and replicate results were omitted from further analyses. Quality of FFPE RNA-derived sequence data was validated as described elsewhere [11].

To detect bimodal expression of genes and exclude potential outliers, SIBER analysis was carried out across the full dataset [22]. The bimodality index of a sample subset was calculated based on the average gene expression level of the subset with the SIBER R package. Normal mixture with Box-Cox transformation mode was used for detection of bimodally expressed genes. Genes with an average read count lower than 25 or genes with counts equal to zero in more than 10% of the samples were excluded to ensure that genes with zero counts across an array of samples were not considered highly bimodal. The resulting table from the outlier analysis was further filtered by excluding genes with mean 1 as zero or BI < 1.3.

### Immunohistochemistry

TMAs consisting of 144 MCC tissue samples were stained for Neurocan protein expression. Sections of 5 μm were cut from the TMA block and processed for immunohistochemistry. Heat-induced epitope retrieval was performed in Dako EnVision™ Flex Target Retrieval solution pH 6.0 (REF K8005, Agilent, CA, USA) with a Biocare Medical Decloaking chamber (Biocare Medical, CA, USA). The TMA slides were blocked with 1% hydrogen peroxide for 30 min, incubated at 4°C overnight with Neurocan antibody (HPA036814, Atlas antibodies, Bromma, Sweden) diluted 1:200 in Draco antibody diluent (cat. no. AD500, WellMed, Duiven, the Netherlands), with Orion detection system (cat. no. R500HRP, WellMed) for 1 h, with 3,3-diaminobenzidine (REF SK4105, Vector Laboratories, CA, USA) for 5 min, and with hematoxylin as counterstain for 1 min. Between incubations, sections were washed with Tris-buffered saline. The samples were digitalized by the Biobank of Helsinki and analyzed with CaseViewer (version 2.3.0.99276, 3DHISTECH, Budapest, Hungary). The staining was frequently heterogeneous in a tumor tissue, and the sample was considered positive when more than 20% of tumor cells expressed NCAN. The samples were divided into four groups based on their expression level in tumor cells (negative, low, intermediate, and high NCAN expression). The stainings were graded by M. Salmikangas. A heart muscle was used as a negative and cerebellum as a positive tissue control in immunohistochemistry.

### Statistics and data analysis

IBM SPSS Statistics 26 was used for statistics and data analyses. Kaplan-Meier and Cox proportional hazards models’ p-values were calculated with log-rank method. The survival time was calculated from the date of diagnosis until one of the two following endpoints: death or end of follow-up on 31 December 2016. Pearson Chi-square or Fisher-Freeman-Halton exact test and Kruskal-Wallis tests were used to analyze the statistical significance of immunohistochemistry results.

## Results

### Bimodally expressed genes in MCC

**Fig 1:**
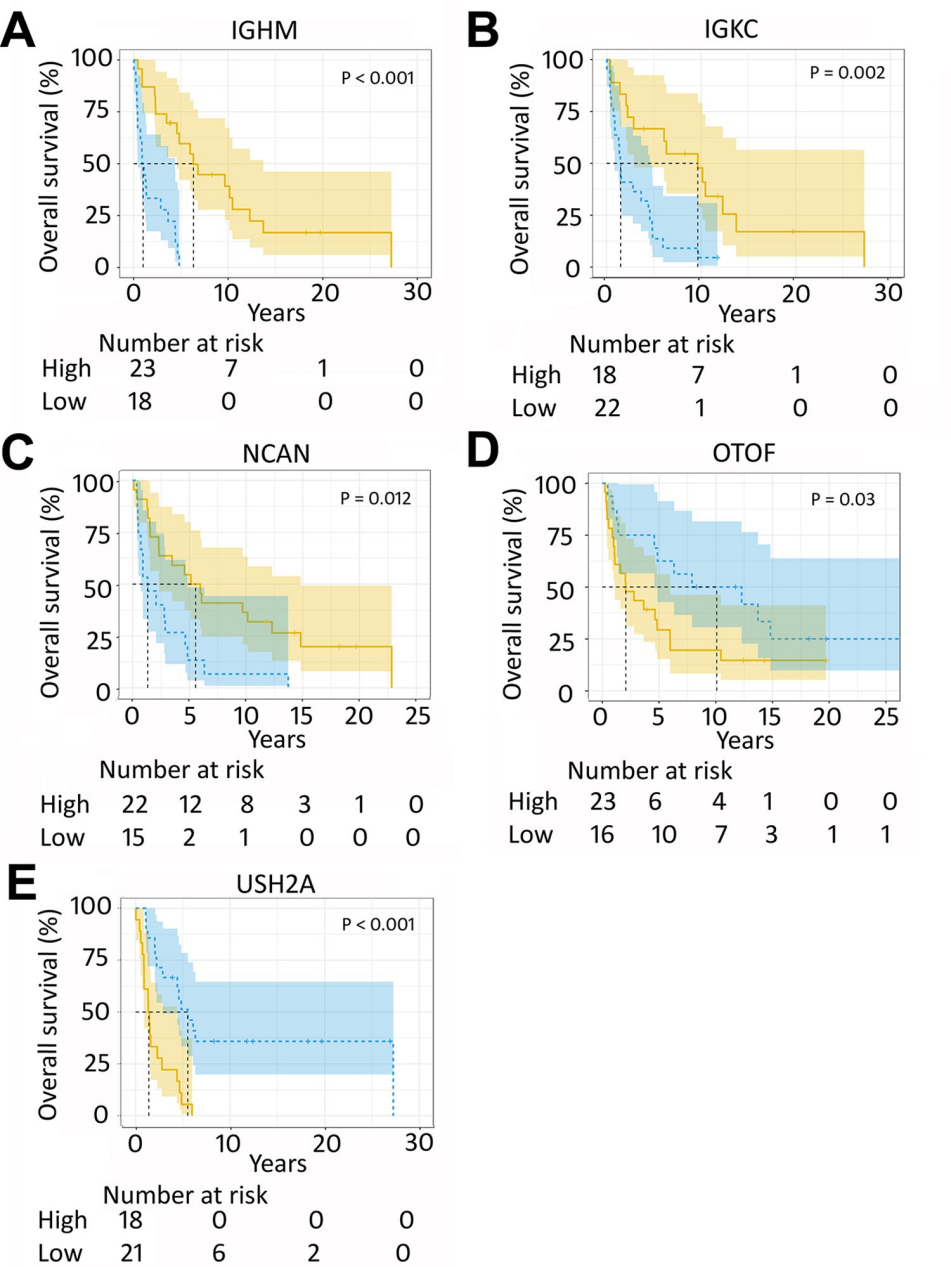
Kaplan-Meier overall survival analyses of bimodally expressed genes *IGHM*, *IGKC*, *NCAN*, *OTOF*, and *USH2A*. Elevated expression of (A) *IGHM*, (B) *IGKC*, and (C) *NCAN* and decreased expression of (D) *OTOF* and (E) *USH2A* associated with better overall survival.

SIBER analysis was conducted to discover bimodally expressed genes among 110 MCC transcriptomes. The analysis identified 19 bimodally expressed genes: *FLG*, *FOXO6*, *GABRB3*, *IGHG1*, *IGHM*, *IGKC*, *NCAN*, *NELL1*, *OTOF*, *PLCB1*, *PMEPA1*, *PRPH*, *RORB*, *SCNN1A*, *SYN2*, *TRDC*, *TRIM29*, *USH2A*, and *XIST* (S1 Table). Kaplan-Meier analysis for overall survival was performed to compare outcome between tumor samples with the highest 25% and the lowest 25% gene expression of each gene. Of the 110 patients, 96 died during follow-up (median follow-up 2.3 years; range 8 days to 27.2 years). Five of the genes (*IGHM*, *IGKC*, *NCAN*, *OTOF*, *and USH2A*) were associated with overall survival (p < 0.05; Fig 1).

### NCAN expression in clinical MCC sample series

**Fig 2:**
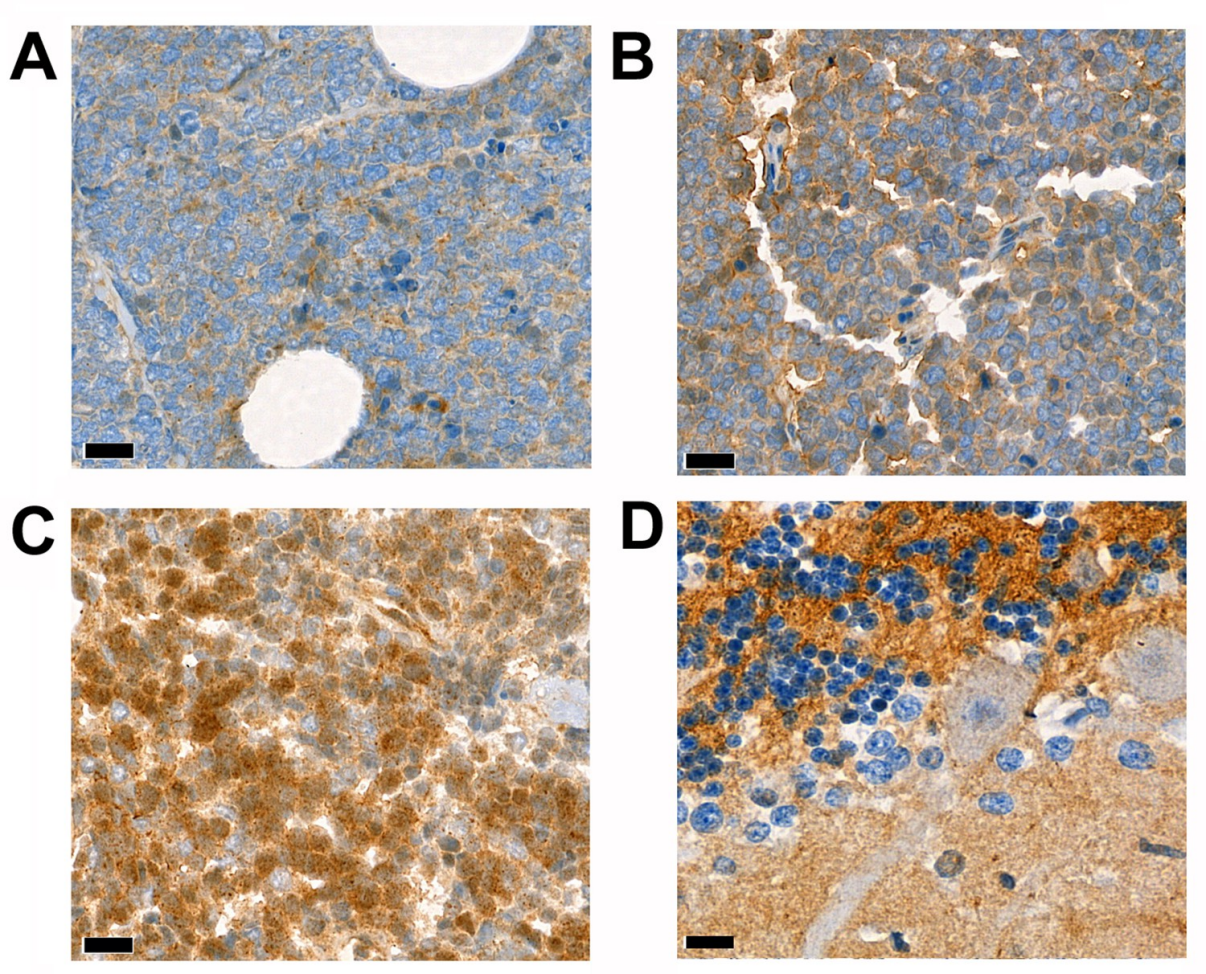
**NCAN expression in tissue samples**. Immunohistochemical staining in representative MCC samples showing (A) low, (B) intermediate, and (C) high NCAN expression. The expression is primary intracytoplasmic. (D) Cerebellum was used as a positive control for NCAN expressing tissue. Scale bar 20 μm.

NCAN protein expression was next investigated in 144 immunostained samples on a tissue microarray. The samples overlapped with all 110 RNA-sequenced samples from which tissue was available for RNA-sequencing and sequencing was successfully conducted. All MCC samples showed NCAN expression (Fig 2). Low, intermediate, or high NCAN expression was detected in 31, 60, and 53 samples, respectively. NCAN expression pattern was homogenous in five whole tissue MCC sections analyzed.

Low NCAN expression was associated with an absence of MCPyV DNA and viral LT antigen expression (p = 0.002; p = 0.004; Table 1). No significant associations were found with sex, age, tumor diameter, primary tumor location, or tumor stage (all p-values > 0.05).

**Table 1:** NCAN expression in 144 Merkel cell carcinoma samples on tissue microarray.

Factor	NCAN	NCAN	NCAN	
	Low	Intermediate	High	p-value
**Sex**				
Female	20 (19.0)	45 (42.9)	40 (38.1)	0.493
Male	11 (28.2)	15 (38.5)	13 (33.3)	
Total (n = 144)	31	60	53	
**Age**				
Mean (range)	79.6 (65–100)	77.0 (35–95)	77.9 (27–95)	0.980
Median	79	80	81	
**Tumor location**				
Head and neck	19 (24.4)	32 (41.0)	27 (34.6)	0.252
Torso	3 (18.8)	10 (62.5)	3 (18.8)	
Upper/Lower limb	9 (18.0)	18 (36.0)	23 (46.0)	
**Tumor diameter (cm)**				
Mean (range)	2.1 (0.5–5.0)	2.1 (0.6–5.0)	2.2 (0.7–8.5)	0.579
Median	2	1.8	1.5	
**Stage^1^**				
I	12 (23.5)	18 (35.3)	21 (41.2)	0.616*
II	13 (35.1)	15 (40.5)	9 (24.3)	
III	4 (26.7)	7 (46.7)	4 (26.7)	
IV	0	1 (50.0)	1 (50.0)	
NA	2	19	18	
**Presence of MCPyV DNA**				
Positive	10 (11.4)	39 (44.3)	39 (44.3)	0.001
Negative	21 (37.5)	21 (37.5)	14 (25.0)	
**LT antigen expression**				
Positive	11 (12.4)	41 (46.1)	37 (41.6)	0.004
Negative	19 (35.8)	19 (35.8)	15 (28.3)	
NA	1	0	1	

^1^ Classification of cancer stages based on American Joint Committee on Cancer (AJCC) 2018 standard. Groups x/A/B are combined, e.g. II, IIa, and IIb are considered as group II.

* Fisher-Freeman-Halton exact test.

**Fig 3:**
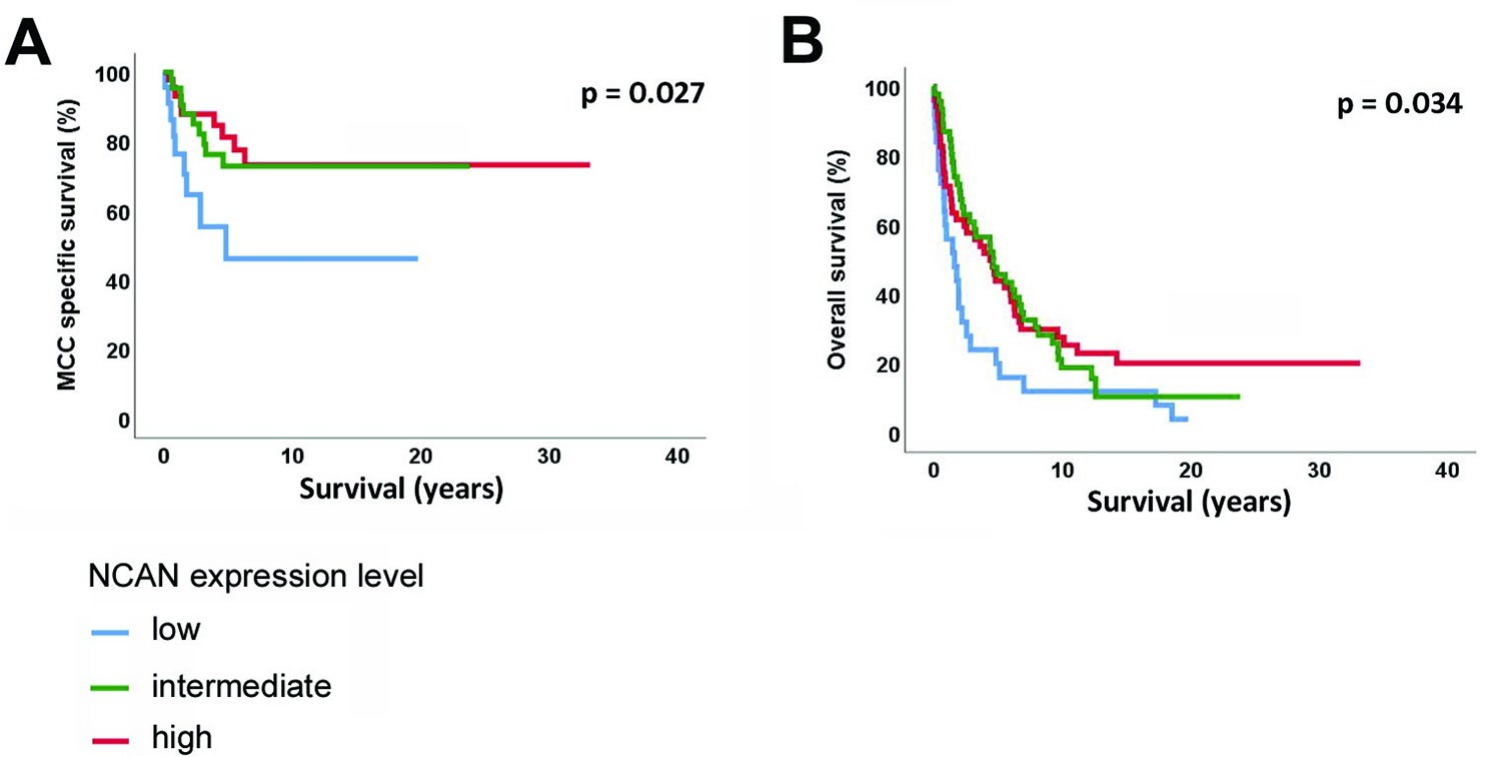
Kaplan-Meier analysis of NCAN expression. (A) MCC-specific survival and (B) overall survival.

During the follow-up123 of 144 patients (85.4%) died from any cause and 41 (28.5%) died from MCC (median follow-up 2.4 years, range 6 days to 33.2 years). Low NCAN expression was associated with worse MCC-specific survival and overall survival (p = 0.027; p = 0.034; Fig 3). However, when presence of MCPyV DNA and NCAN expression (low vs. intermediate and high) were investigated in a multivariable Cox proportional hazards analysis for MCC-specific survival, virus-negativity was associated with a significantly worse outcome (Hazard ratio [HR] = 2.29; 95% confidence interval [95% CI] = 1.22 to 4.33; p = 0.010), whereas low NCAN expression only showed a tendency for worse outcome (HR = 1.84; 95% CI = 0.91 to 3.70; p = 0.087).

## Discussion

In this study, we discovered five genes, *IGHM*, *IGKC*, *NCAN*, *OTOF*, *and USH2A*, that were bimodally expressed and whose expression was associated with overall survival in transcriptome data of 110 Finnish MCCs. When NCAN expression was assessed with immunohistochemistry, all MCC samples expressed the protein, and increased expression was associated with MCPyV-positivity of tumors and better overall and disease-specific survival. However, when virus status was taken into account in Cox multivariate analysis, NCAN expression showed only a tendency for better MCC-specific disease outcome. The results indicate that increased NCAN expression is not an independent prognostic factor in MCC. Instead, NCAN is related to VP-MCC phenotype that have better outcome. However, the frequent expression of NCAN in MCC and its strong association with the presence of MCPyV warrants further research of its in MCC tumorigenesis.

NCAN belongs to group of chondroitin sulphate proteoglycans, which are structural components of the extracellular matrix and have a role in neuronal growth mechanisms of axon guidance, neural plasticity or neural repair processes following injury of the spinal cord or brain [23]. Contrary to our finding in MCC, NCAN expression has earlier been associated with poor outcome and progression in cancer. NCAN has been associated with poor outcome in neuroblastoma, with NCAN promoting anchorage-independent growth and chemoresistance of neuroblastoma cells [24]. NCAN expression promotes also glioma cell proliferation and invasion through the Rho/Rho-associated protein kinase pathway [25,26]. Hypoxia plays a role in induction of NCAN expression in the nervous system. Increased NCAN levels have been detected in a retinal ischemia model in the rat [27]. In addition, NCAN secretion is increased in conditioned medium from oxygen/glucose-deprived neural stem cells [26]. Better outcome in NCAN-positive MCCs might be explained by the association with MCPyV-positive tumors, which in part associated with better survival in MCC [2–4]. Whether MCPyV- and NCAN-positive tumors share tumorigenic mechanisms and signaling pathways warrants further *in vitro* and *in vivo* studies in MCC models.

We investigated MCC for bimodally expressed genes because several studies have shown bimodal genome, transcriptome and methylome patterns in cancer when MCPyV-negative and positive tumors are compared to each other [4,6,7,19,20]. Bimodal genes can be defined as having two modes of expression within the same population, and bimodal gene expression analyses have been used to discover genes that play an important role in cancer prognosis, progression, or therapy responses [28–30]. Estrogen receptor (*ESR1*) expression in breast cancer is a good example of a gene, whose expression has both prognostic and treatment defining roles in cancer [28]. We used the SIBER method to identify bimodally expressed genes in MCC as it has been shown to be suitable to analyze RNAseq data [22].

We conducted RNA sequencing with tumor tissue sections, which contain various cell types and therefore, the results reflect more tissue than tumor cell level expression patterns. Although, immunoglobulin genes are reported to be expressed in MCC [31], we can’t rule out the possibility that *IGHM* and *IGKC* expression signal originated from immune cells. *IGKC* and *IGHM* associated with a better overall survival that is also seen with MCCs with a strong immune cell infiltration [4,9–13]. We used FFPE tissue samples to extract RNA and degradation nucleic acids in formalin-fixed tissues are known to produce a poor RNA sample quality. However, we have showed earlier with the data set that 3’ tag counting and QuantSeq method produce similar transcriptome profiles when compared with the profiles produced from frozen tissue material by Affymetrix gene expression array [11].

To conclude, we discovered that NCAN is frequently expressed in MCC, and its expression is associated with presence of MCPyV in tumor cells and improved disease outcome. Further studies are required to investigate the role of NCAN in MCC tumorigenesis.

## Supporting information

S1 Table Results of the systematic identification of bimodally expressed genes analysis using RNAseq data (SIBER). Data was derived from 110 Merkel cell carcinoma tissue samples.
(PDF)
