# Supplementary material for: Neurocan expression associates with better survival and viral positivity in Merkel cell carcinoma
Source: PLoS One. 2023 May 5;18(5):e0285524. doi: 10.1371/journal.pone.0285524 (PMC10162530; doi:10.1371/journal.pone.0285524)
Supplement: S1 Table — Data was derived from 110 Merkel cell carcinoma tissue samples. (PDF) [file pone.0285524.s001.pdf]

Gene_name	mu1	mu2	sigma1	sigma2	pi1	delta	BI
FLG	3.17092206993683	5.77092077262474	0.871606002393881	0.871606002393881	0.594734486432813	2.98299770257085	1.46448289781431
FOXO6	3.05693748890174	5.38589553464823	0.856503470487824	0.856503470487824	0.493550452347016	2.71914607003287	1.35945992290319
GABRB3	2.76451425879404	4.88565379677302	0.759477406002316	0.759477406002316	0.392477728990112	2.79289353602247	1.36377583697739
IGHG1	3.06123279805849	6.17128390037066	1.05025292485911	1.05025292485911	0.692842095390642	2.96124012482932	1.36606576244729
IGHM	3.17051686871719	5.78632710162198	0.97607836343093	0.97607836343093	0.587841162054816	2.67991826364246	1.31911862847212
IGKC	3.79405932358195	7.34135488457614	1.29815113000188	1.29815113000188	0.641293955564652	2.73257518251287	1.31059962825584
NCAN	3.00956787293427	5.43800512703047	0.909962399459149	0.909962399459149	0.512042387811854	2.66872263682497	1.33397424650534
NELL1	2.70899594909686	4.63992636696865	0.712524526663486	0.712524526663486	0.613538389591345	2.70998449262327	1.31959559271015
OTOF	3.20101118675862	6.10660053841192	0.900327425666596	0.900327425666596	0.743315089682727	3.22725851598047	1.40967954967541
PLCB1	3.09323111074588	5.2141576853651	0.757702294462717	0.757702294462717	0.602966166521306	2.79915553921235	1.36957954710974
PMEPA1	3.09918852649482	5.3009630431718	0.783144472694485	0.783144472694485	0.320940127919638	2.81145381656282	1.31249300697383
PRPH	2.66369496651863	4.72656346418042	0.658868008091853	0.658868008091853	0.73137204722013	3.13092830783523	1.3877713048102
RORB	3.04011960468547	5.7915296815336	0.914287421902104	0.914287421902104	0.387559534782989	3.0093491509749	1.46613421934808
SCNN1A	2.98355917730481	5.50360914777816	0.912815167667519	0.912815167667519	0.478274946580385	2.76074506618106	1.37906890673096
SYN2	3.16683438657446	5.68558477693274	0.814203635684791	0.814203635684791	0.420822906501376	3.09351405467487	1.52724062485044
TRDC	3.08804146356803	5.94514882651101	1.01384568742344	1.01384568742344	0.629105763159863	2.81808898374265	1.3612615467471
TRIM29	3.41516679446904	5.90507202610759	0.941630822275236	0.941630822275236	0.483924068461986	2.6442478015134	1.3214403563583
USH2A	2.94161954273779	6.53925871328666	1.05598781916346	1.05598781916346	0.312737601205247	3.40689457327156	1.57946510907036
XIST	3.29138915812351	8.14558274652592	1.10005212790962	1.10005212790962	0.278815502277163	4.4126941489824	1.97872516351039
